# Assessment of social support in HIV-positive individuals attending a tertiary care center

**DOI:** 10.4103/2589-0557.69007

**Published:** 2010

**Authors:** Debashis Nath, Naveet Wig, Nitin Mishra, Sajan Jiv Singh Nagpal, Madhu Vajpayee, Hemraj Pal, C. S. Pandav

**Affiliations:** Department of Medicine, All India Institute of Medical Sciences, New Delhi, India; 1 Department of Microbiology, All India Institute of Medical Sciences, New Delhi, India; 2 Department of Psychiatry, All India Institute of Medical Sciences, New Delhi, India; 3 Center for Community Medicine, All India Institute of Medical Sciences, New Delhi, India

Sir,

Adult prevalence of human immunodeficiency virus (HIV) in India is 0.36%, amounting to approximately 2.5 million people living with HIV and acquired immunodeficiency syndrome.[1] Since the availability of Highly Active Anti-Retroviral Therapy HAART, improvement in quality of life (QOL) of patients has become a key issue in patient management.[2] A higher level of social support improves mental health, provides more days of vitality and, most importantly, better social support has been found to be associated with improved QOL.[3] In India, where 72.2% of the population lives in rural areas,[4] with joint families being more common than nuclear ones, it may prove to be a valuable tool along with HAART in the management of HIV-positive individuals. We present assessment of social support in such patients.

We studied a total of 82 HIV-positive individuals who were older than 18 years of age and who gave informed written consent. Of the 82 patients studied, 80% were males.

Social support score were assigned based on Dube’s scale.^[5]^ The scale consisted of 34 items divided into four domains of: (1) social network eleven items), (2) financial support (eight items), (3) emotional support (ten items), (4) belief support (five items). Each item was scored on a five-point rating scale. Thus, four individual dimension scores were obtained by simply adding the item responses for each dimension. The greater the score, higher is the value of total social support. Because different dimensions of social support had different numbers of items and hence different mean scores, a transformed scored from 0 to 100 was obtained for comparison by dividing the difference between the mean value and the minimum possible value for the given dimension by the difference between the maximum and minimum possible value for the dimension.

The social support score was highest for belief support (87.65), followed by social network (55.38), emotional support (54.34) and financial support (54.25). Social support scores for the four dimensions are tabulated in Table 1.

**Table 1:** Social support scores for HIV positive individuals

Social support dimensions	Possible values		Observed values		Range (minimum possible value-maximum possible value)	Mean	Std. deviation	Transformed score
	Minimum	Maximum	Minimum	Maximum				
Social network	11	55	19	49	44	35.37	7.28	55.38
Financial support	8	40	11	39	32	25.39	7.06	54.34
Emotional support	10	50	14	48	40	31.70	7.80	54.25
Belief support	5	25	5	25	20	17.53	5.86	87.65
Total Social support	34	170	69	149	136	110.01	21.71	55.88

The belief support score was found to be considerably higher than other dimensions. Belief support shows patient’s faith in God/religious guru, treating physician, nursing care and drugs/medical facilities. The highly religious nature of Indians with considerable faith in God and the treating physician can explain this result.

The belief support score only assessed patients’ own faith and beliefs while all other domains’ score also depended on contribution from family members/friends/relatives. This reflects the influence of stigma attached to the disease. Education of significant others in patients’ life is therefore required to provide them with better social support and improved QOL.
